# Electrical Conductivity Measurement in Human Liver Tissue: Assessment on Normal vs. Tumor Tissue and under In Vivo vs. Ex Vivo Conditions

**DOI:** 10.3390/bios14080382

**Published:** 2024-08-08

**Authors:** Amirhossein Sarreshtehdari, Tomás García-Sánchez, Patricia Sánchez-Velázquez, Benedetto Ielpo, Enrique Berjano, María Villamonte, Xavier Moll, Fernando Burdio

**Affiliations:** 1 Department of Experimental and Health Sciences, Hospital del Mar Medical Research Institute (IMIM), Universitat Pompeu Fabra, 08005 Barcelona, Spain; amirhsar@gmail.com (A.S.); maryvr31@hotmail.com (M.V.); 2 Department of In formation and Communication Technologies, Universitat Pompeu Fabra, 08018 Barcelona, Spain; tomas.garcia@upf.edu; 3 General Surgery Department, Hospital del Mar Medical Research Institute (IMIM), Universitat Pompeu Fabra, 08005 Barcelona, Spain; patri_sv5@hotmail.com (P.S.-V.); ielpo.b@gmail.com (B.I.); 4 BioMIT, Department of Electronic Engineering, Universitat Politècnica de València, 46022 Valencia, Spain; eberjano@eln.upv.es; 5 Departament de Medicina i Cirurgia Animals, Facultat de Veterinària, Universitat Autònoma de Barcelona, 08193 Barcelona, Spain; xavier.moll@uab.cat; 6 Fundació Hospital Clínic Veterinari, Universitat Autònoma de Barcelona, 08193 Bellaterra, Spain

**Keywords:** electrical conductivity, ex vivo, human liver, in vivo, tumor

## Abstract

Background: This study evaluated electrical conductivity in human liver tissue in the 3–1000 kHz frequency range to compare normal versus tumor tissues under in vivo versus ex vivo conditions. Methods: Previous informed consent was obtained from twenty patients undergoing liver resection in whom liver electrical conductivity was measured during surgery and after resection. Result: We found higher electrical conductivity values in tumor tissues than in normal tissue in both in vivo (0.41 ± 0.10 vs. 0.13 ± 0.06 S/m) and ex vivo (0.27 ± 0.09 vs. 0.12 ± 0.07 S/m) conditions (at 3 kHz). The electric properties also showed a promising potential for distinguishing between different tissue types including metastasis, cholangiocarcinoma (CCA), hepatocellular carcinoma (HCC), hepatic cirrhosis, and normal liver (both in vivo and ex vivo). At 3 kHz, in vivo electrical conductivity for cholangiocarcinoma, HCC, and metastasis were 0.35, 0.42 ± 0.13, and 0.41 ± 0.08 S/m, respectively, which differed significantly from each other (*p* < 0.05). Conclusions: These findings could potentially improve liver disease diagnostics through electrical conductivity measurements and treatment techniques involving electric fields. Future research should focus on expanding the sample size to refine the categorization and comparison processes across diverse human liver tissue types.

## 1. Introduction

In 2020, colorectal cancer liver metastasis was projected to be the second most prevalent cancer diagnosis and the second major cause of cancer fatalities in Europe, with close to 520,000 new cases and 245,000 deaths per year [1]. Surgical resection continues to be a favored method of treatment [2], while ablative therapy based on radiofrequency (RF) and irreversible electroporation (IRE) (positioning electrodes in the vicinity of the tumor) are also valuable treatment methods [3,4]. Implementing RF thermal ablation requires a comprehensive understanding of the tissues’ electrical properties [5]. Innovate IRE-based ablation therapies also require a precise characterization of the electrical properties of tumors and normal tissues [6], which have also shown potential in the diagnostic detection of tumors [7]. Biological tissues, such as hepatic tissue, are known to exhibit frequency-dependent electrical properties, a phenomenon that is crucial in the field of bioimpedance analysis [8]. This means that these tissues’ electrical bioimpedance changes with the frequency of the electrical current applied, which can be used to explore the tissue’s structure and function [7,8]. The characteristics of liver tissue, including electrical conductivity, are thus important for determining exactly how this tissue interacts with electromagnetic fields. This information can also be used for developing therapies and advancing physics-based research on the properties of biological tissues. Many research groups have focused on the study of biological tissues’ electrical properties [9]. For instance, Gabriel et al. made a notable contribution by methodically examining the dielectric characteristics of many animal and human ex vivo tissues [10]; however, not enough research has been carried out so far to link these disparities to species differences.

In previous studies on the application of IRE (a method that causes cell death by creating irreversible pores in the cell membranes due to the application of short and high-intensity electrical pulses), our goal was to alter the basal electrical properties of normal and tumor tissue to open a therapeutic window to improve treatment outcomes [11,12,13]. In particular, we demonstrated the potential of altering tumor conductivity by infusing a low-conductivity solution into the common artery or increasing the conductivity of normal tissue by infusing a high-conductivity solution into the hepatic vein. Our present objective is to assess variations in electrical conductivity between tumor and normal human liver tissue. The available current literature on the electrical properties of liver tissue is mainly based on animals or ex vivo human tissues, with the only exception, to our knowledge, of the recent study by Halonen et al. [14], who measured electrical impedance in tumor and normal liver tissue in humans by means of open-ended coaxial probe installed on a biopsy needle, from 1 kHz to 349 kHz. In contrast, our objective was to obtain the values of electrical conductivity of tumor and normal tissue liver up to 1 MHz and compare the ex vivo and in vivo values.

## 2. Materials and Methods

This study was approved by the Ethics Committee on Biomedical Research (CPMP/ICH/135/95) and the IRB (CEImPSMAR_2020-9026-I) of Consorci Mar Parc de Salut de Barcelona (Barcelona, Spain). Both the open and laparoscopic surgical procedures and the bioimpedance electrical measurements proceeded according to Law 14/2007 on Biomedical Research, Ethical Principles of the Declaration of Helsinki.

### 2.1. Human Subjects

After giving their informed consent for the measurements and the use of their data for analysis, a total of 20 patients were chosen for this study, with ages ranging from 50 to 86 years of age (12 men and 8 women). Since each patient had a unique diagnosis, we included a diverse group of malignancies. Three of these patients were also found to have cirrhosis, a disease that can affect liver electrical conductivity. The purpose of the patient selection process was to increase the applicability of our findings by including a wide range of diseases and factors.

### 2.2. Electrical Bioimpedance Measurement

**Figure 1:**
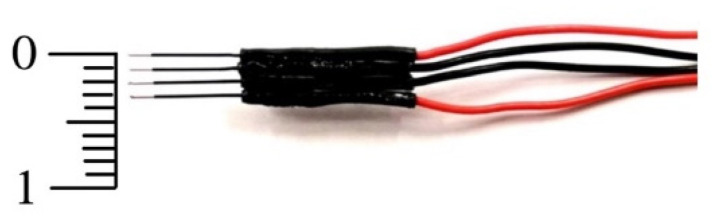
Arrangement of in-line, four-needle electrodes used to measure electrical impedance. The two outermost electrodes injected the electrical current, while the two central electrodes picked up the electrical voltage (scale in mm).

A four-electrode probe was assembled with two pairs of SDN Model RD/BK 12/1500, 90° electrodes (inomed Medizintechnik GmbH, Emmendingen, Germany). These electrodes contained stainless steel needles with an outer diameter of 0.35 mm and a total length of 12 mm. Only the last 3 mm at the tip of the needles were electrically active, while the rest of the needle was electrically isolated. Four needles were mounted in parallel using a biocompatible LOCTITE^®^ 431 instant adhesive (Henkel, Düsseldorf, Germany). The distance between the electrodes was 1 mm (see Figure 1). This single-use arrangement was used in all the impedance measurements.

**Figure 2:**
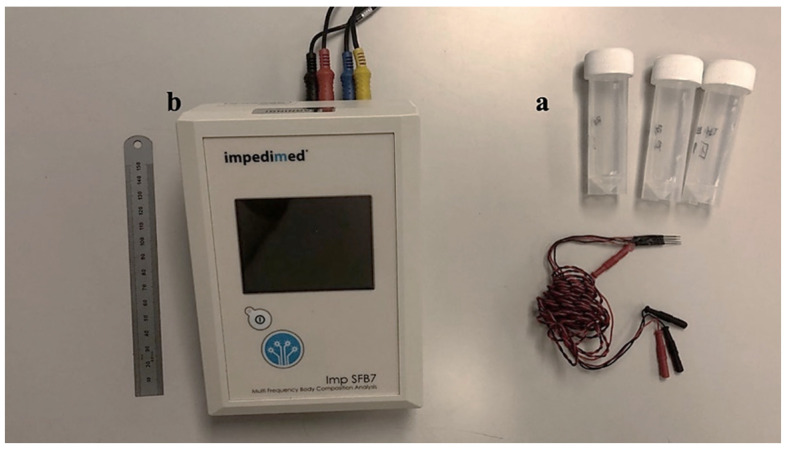
Material and devices used for impedance measurement in tissue. (**a**) Reference buffers (saline solution) with different conductivities. (**b**) Bioimpedance analyzer SFB7 from ImpediMed (Carlsbad, CA, USA).

Three saline buffer solutions were prepared using NaCl and distilled water. The electrical conductivity of these solutions (referred to hereinafter as Reference #1, Reference #2, and Reference #3) was measured with a conductometer resulting in average values reported at 20 °C, of 0.062 S/m, 0.13 S/m, and 0.25 S/m, respectively. A calibration procedure was used to lessen the effects of the measurement system, especially those that affected the high and low-frequency readings (stray capacitances and inductive effects). This calibration functioned as a compensatory measure under the assumption that the frequency spectrum of the buffer solutions should be flat, in contrast to that shown in biological tissues. This calibration procedure also allowed the conversion from impedance (Z) to conductivity (σ). Only the impedance spectrum of saline buffer Reference #3 was used for calibration since its impedance was closer to the tissue low-frequency impedance measured. This method guaranteed the precision and dependability of our results. The SFB7 equipment produced by ImpediMed (Carlsbad, CA, USA), originally intended for clinical and research settings to detect tissue composition and fluid status, was used to measure electrical impedance by the tetrapolar mode (see Figure 2). This device scans 256 distinct frequencies from 3 kHz to 1000 kHz.

### 2.3. Measurement Procedure

**Figure 3:**
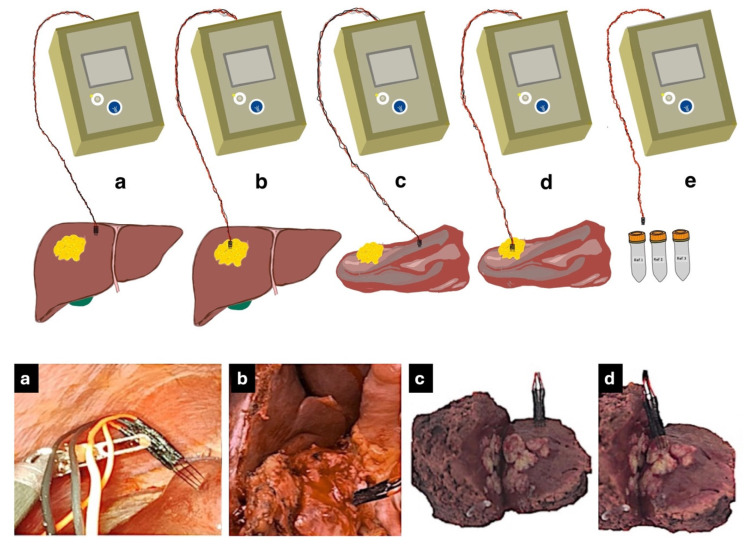
Roadmap of measurement procedure. (**a**) Measuring bioimpedance in normal liver tissue in vivo by laparoscopic approach. (**b**) Measuring bioimpedance in tumor tissue in vivo. (**c**) Measuring bioimpedance in dissected normal tissue ex vivo. (**d**) Measuring bioimpedance in dissected tumor tissue ex vivo. (**e**) Measuring bioimpedance with reference buffers #1, #2, and #3.

Following the surgical team’s access to the liver tissue, sterilized electrodes were inserted into the liver, initially into normal tissue to avoid contamination from tumor tissue. They were then inserted into the tumor tissue and impedance was measured again. The impedance of both the normal and tumor tissues was measured ex vivo after surgical resection. These steps are shown in Figure 3. The average temperature of the in vivo tissue was 36 °C since they were taken mainly in liver tissue during laparoscopic surgery. The temperature of the ex vivo measurements was 20 °C (surgery theatre ambient temperature) since they were taken in the dissected pieces immediately after extraction until the impedance was measured (10 ± 2 min).

### 2.4. Electrical Conductivity Analysis

Data analysis was conducted on MATLAB software Version R2021b (MathWorks, Natick, MA, USA) to convert impedance data into electrical conductivity (measured in S/m). This process involved the calibration of impedance data using reference solution measurements and then calculating conductivity data. Electrical conductivity (σ) was calculated using the real part and the cell constant k, as follows:

The values of the real (R) and imaginary part (X) of the measured impedance Z for each of the 256 frequencies were used to calculate the conductance G at each frequency, as follows:

(1) $$G=\frac{R}{R^{2}+X^{2}}$$

G is the real part of the admittance Y (Z = 1/Y). We used Equation (1) to calculate the values of G for each frequency in the case of buffer Reference #3 (i.e., G_buffer_). Next, since we previously measured the electrical conductivity of this buffer (σ = 0.25 S/m) and its value is expected not to change with frequency, we used Equation (2) to calculate the conversion factor *k* that relates the value of G_buffer_(f) to the electrical conductivity of the buffer:

(2) $$k(f)=\frac{\sigma }{G_{buffer}(f)}=\frac{0.25}{G_{buffer}(f)}$$

Finally, the k(f) values calculated for each frequency and the values of conductance measured for each tissue and frequency G_tissue_ were used to obtain the electrical conductivity for each tissue and for each frequency as follows:

(3) $$\sigma_{tissue}(f)=k\left(f\right)\cdot G_{tissue}(f)$$

For simplicity, five different frequencies (3 kHz, 30 kHz, 300 kHz, 607 kHz, and 1000 kHz) were chosen for detailed analysis. These frequencies were selected from among 256 different frequencies in a range from 3 kHz to 1000 kHz since they are potentially of interest for different diagnostic and therapeutic applications.

### 2.5. Statistical Analyses

All the statistical analyses were conducted on SPSS Statistical Software Version 21 (IBM, Armonk, NY, USA). Normality was tested by the Shapiro-Wilk statistic. The mean and standard deviation of the conductivities were compared by a non-parametric Kruskal–Wallis test for the electric values at frequencies of 3 kHz and 30 kHz, 300 kHz, 607 kHz, and 1 MHz. A P-value less than 0.05 was considered statistically significant. Due to the small number of cases, a non-parametric test (Kruskal–Wallis test) was also conducted for cirrhotic tissue.

## 3. Results

### 3.1. Impedance Calibration and Calculation

**Figure 4:**
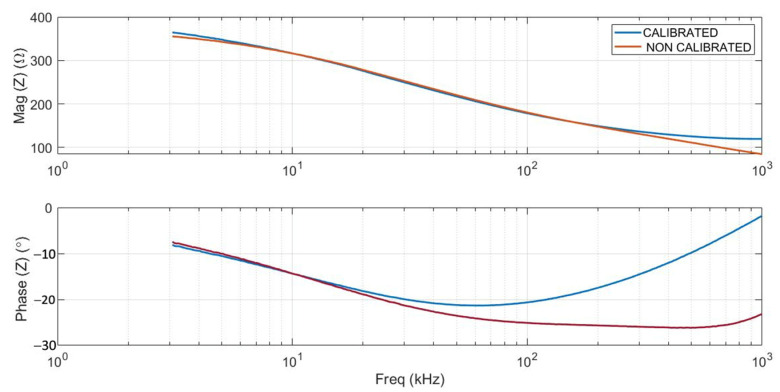
Example of calibrated (blue lines) and non-calibrated (red lines) impedance data. Magnitude and phase spectra of normal liver tissue are shown.

Figure 4 shows an example of the electrical impedance values (magnitude and phase) measured across the entire spectrum considered (256 points within the range 3 kHz–1 MHz) before and after calibration, using the reference buffer. It can be clearly seen that the calibration corrected the high-frequency deviations caused by capacitance and parasitic inductance.

### 3.2. Conductivity Measured in Normal and Tumor Tissue (In Vivo and Ex Vivo)

Five frequencies were chosen to calculate electrical conductivity for four different situations: in vivo and ex vivo tumor, and in vivo and ex vivo normal tissue. Although data were collected from 20 patients during surgery, only 19 were included in the analysis because technical problems invalidated the records of one patient (the conductivity of tumor tissue in vivo, only 15 patients’ data were available for the same reason), and so forth. Table 1 shows the tissue electrical conductivity mean and standard deviation at the chosen frequencies. As the results of all the frequencies from 3 kHz to 1000 kHz followed a non-normal distribution, a Kruskal–Wallis test was applied, which indicated a significant difference (*p* < 0.05) in electrical conductivity values across all the types of tissues at these frequencies. The in vivo tumor tissue had the highest conductivity at all frequencies. For example, at 3 kHz, the conductivity of in vivo tumor tissue was found to be 0.41 ± 0.10 S/m followed by the ex vivo tumor tissue with 0.27 ± 0.09 S/m. Ex vivo normal tissue had the lowest conductivity, measuring 0.12 ± 0.07 S/m, while in vivo normal tissue offered a value of 0.13 ± 0.06 S/m.

### 3.3. Electrical Conductivity Ratio

**Figure 5:**
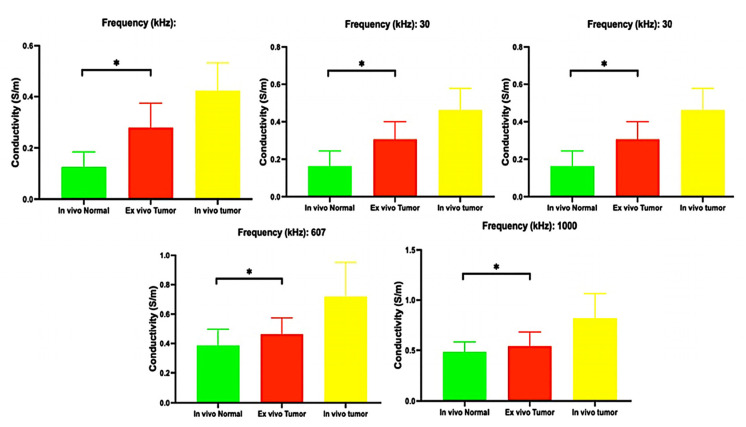
Mean and standard deviation of electrical conductivities (*: Significative differences with *p* < 0.05).

The differences in electrical conductivity values between various types of tissues are shown in Table 2, along with the ratios, to compare (1) in vivo tumor vs. normal tissue; (2) tumor tissue in vivo vs. ex vivo; (3) tumor tissue ex vivo vs. normal in vivo; (4) in vivo tumor vs. cirrhotic tissue; (5) ex vivo tumor vs. cirrhotic tissue and (6) in vivo normal vs. cirrhotic tissue. Figure 5 gives the electrical conductivities for the five chosen frequencies. The difference between the frequencies was statistically significant (*p* < 0.05).

### 3.4. Differences between Normal Tissue Conductivity and Cirrhosis In Vivo and Ex Vivo

In the non-parametric analysis, we found a significant difference (*p* < 0.05) between normal in vivo tissue, tumor in vivo, and cirrhotic in vivo tissue at all chosen frequencies. The results for cirrhotic tissue are shown in Table 1.

### 3.5. Tissue Conductivity Based on Tumor Type and Status

Table 3 and Table 4 show the mean and standard deviation of electrical conductivity for tumor tissue in vivo and ex vivo respectively in three different tumor types, classified into three categories: metastasis, cholangiocarcinoma (CCA), and hepatocellular carcinoma (HCC). Each category was characterized by its mean and standard deviation of electrical conductivity, providing an overview of the electrical properties across different tumor types.

### 3.6. Supplementary Information on Case-by-Case Conductivity at a Frequency 1 MHz

Table 5 shows an example of the patients’ tissue conductivity at 1000 kHz, including the variables of the tissue type (malignant or normal), tissue state (in vivo or ex vivo), and extra contextual elements like tumor size and whether or not the normal tissue showed cirrhotic or non-cirrhotic features. It is important to remember that, as already mentioned, technical constraints made it difficult to evaluate conductivity in some cases.

## 4. Discussion

This is the first study to provide electrical conductivity measurements in vivo and ex vivo human liver tissue of the normal and malignant forms in the frequency range between 3 kHz and 1 MHz. We also provide human in vivo data to improve current cancer ablation technologies, notably irreversible electroporation (IRE). When it comes to treating scattered liver tumors, the effectiveness of therapeutic interventions largely depends on the natural electrical properties of the tissue. Although needle puncture is currently the most common method for IRE procedures, it is important to note that future developments, such as non-discriminatory electrodes or plates, could make this technique even more valuable. The measurement method used in this study was safe, straightforward, and quick, making it appropriate for replication in similar studies with minimally invasive procedures in the operating theatre.

The findings reveal significant variations in conductivity across all types of tissue at all frequencies (*p* < 0.05) and align with previous research findings [9,15,16]. The differences in electrical conductivity between tumor and normal liver tissue are also consistent with previous results in other studies [8,15,16]. As can be seen in Table 2, the ratio of conductivity shows that in vivo tumor tissue has higher conductivity than normal tissue. This variation may be attributable to the differences in angiogenesis between tumor and normal tissues. Numerous studies suggest that the portal vein in normal tissue accounts for 70% of the blood flow, while the hepatic artery contributes the remaining 30%. In contrast, in a tumor, the blood supply is primarily derived from the hepatic artery [17,18,19,20]. Furthermore, living tissues typically rely on a steady metabolic process, which involves the release of metabolic molecules facilitated by the absorption of nutrients, energy, and oxygen through the capillary network. The exchange of ions helps maintain osmotic pressure in the cell membrane. Conversely, in dissected tissue they lose their oxygen and blood supply, leading to changes in the permeability of the cell membrane and fluctuations in ion concentrations within and outside the cell. These adjustments ultimately modify the tissue’s dielectric characteristics [9].

Interestingly, the conductivity ratio between an ex vivo tumor and its corresponding value of in vivo normal tissue was usually much lower than the ratio between the in vivo tumor and in vivo normal tissue (Table 2). As shown in Table 5 in five patients (10, 12, 13, 14, 15), the ex vivo conductivity in tumor tissue has a greater value than the corresponding value in vivo in normal tissue. That may be particularly interesting since the ex vivo status of the tumor could be similar to a “non-perfusion status” when the hepatic artery is temporally clamped (given that the single source of blood perfusion in tumor tissue is the hepatic artery). The hepatic artery can be clamped via laparotomy or minimally invasive approaches, such as laparoscopy or percutaneous methods. Considering the predicted increase in impedance with glucose solution 5% via intraarterial infusion (mean maximum value of 4.7 times) that was shown in our previous study in an animal model [21], the ratio mentioned above of conductivity between tumors versus their corresponding values in normal tissue (usually less than 2.7 times) and the inverse proportional relationship between conductivity and impedance make it easy to predict selectivity of action in tumor tissue when IRE is applied. This could be especially true if the best scenario is selected (e.g., high frequencies and arterial clamping).

Our results have potentially important implications for therapeutic and diagnostic techniques such as radiofrequency ablation (RF) [22,23] and irreversible electroporation (IRE) [24,25]. These techniques could potentially be optimized by leveraging the unique electrical characteristics of various tissue types, while our electrical impedance data could be exploited in future electromagnetic computer simulation studies that require real data to build the characteristics of living tissues. In this context, machine learning methods can potentially be very useful in identifying patterns in the bioimpedance of healthy and pathologic tissues [26].

The electrical conductivity of cirrhotic and normal tissue was found to differ significantly at all frequencies. Cirrhotic tissue had an electrical conductivity of 0.09 to 0.01 to 0.04 to 0.01 S/m at a frequency from 3 at 1000 kHz, while in non-cirrhotic tissue it was 0.13 to 0.49 S/m. This finding agrees with that reported by O’Rourke et al. [16] who found that the electrical conductivity of cirrhotic liver tissue was higher than that of normal tissue (measured on 11 patients): 1.38 ± 0.15 vs. 1.16 ± 0.14 S/m at 950 MHz, and 2.21 ± 0.17 vs. 1.95 ± 0.18 S/m at 2.45 GHz. While these contrasting results exist, it is essential to consider the specific context such as different frequencies, measurement techniques, and sample sizes in each study. Further research is needed to fully understand the underlying mechanisms and validate these findings.

Our results show how disconnection from the blood flow resulted in reduced electrical conductivity in both ex vivo tissues. The mean conductivity in tumor tissue and normal tissue ex vivo at 3 kHz, were 0.27 ± 0.04 and 0.12 ± 0.07 s/m, respectively. These differences are aligned with the findings of many other research teams [9,15,16,27,28]. Interestingly, as the frequency increased, the ratio between tissues decreased. As shown in Table 3 and Table 4, the electrical conductivity of cholangiocarcinoma, hepatocellular carcinoma (HCC), and metastasis tumor types was examined. For instance, at a frequency of 3 kHz, the conductivity measurements for cholangiocarcinoma, HCC, and metastasis (in vivo) were found to be 0.35 (one case), 0.42 ± 0.13 and 0.41 ± 0.08 S/m, respectively. This result could potentially offer a glimpse into the complex behavior of tumors. However, it is important to note that these are preliminary findings and further research is certainly needed to validate these results and explore their clinical implications.

These limitations should be taken into account in this study. First, from an electrical perspective, the various tissues under investigation were deemed to be isotropic. Although there has been evidence of anisotropy in hepatic tissue concerning echogenicity [29], anisotropy in the liver is not reported in the major literature on bioimpedance studies throughout a broad frequency range [30,31]. Measuring anisotropy in electrical properties only applies to a few tissues, such as bone and muscle (skeletal and cardiac), where the orientation of the electrodes with respect to the major axis of the tissue (e.g., longitudinal, transversal, or a combination of both) must be precisely assessed [32]. These factors led to the exclusion of anisotropy in bioimpedance measurements from our investigation. Secondly, the measurements collected might have been affected by the sample sizes. Essentially, there must be enough tissue surrounding the electrodes for there to be no errors caused by the presence of other tissues. Using computational modeling, it is possible to estimate the minimal tissue size (see e.g., [33,34]). An alternative method could involve conducting an analytical estimation of the electric field reduction surrounding needle-type electrodes, such as the ones utilized in the current study. The electric field surrounding an electrode with an infinity-long cylindrical geometry reduces proportionally to E(r) ∝ r_0_/r, where r is the radial component of the electric field and r_0_ is the electrode radius [35]. In the present situation, r_0_ = 175 μm indicates that, at a distance of 3.5 mm from the electrode surface, the electric field dropped to 5% of its initial magnitude. Tumor tissue was the most size-limiting tissue because healthy tissue samples, both in vivo and ex vivo, consistently occupied a much larger volume than the tumor tissue. In this regard, the tumor was usually always at least 10 mm in size (see Table 5). This indicates that the boundary of the tumor was, in the worst case, at least 3.5 mm from the electrode surface when the four in-line electrodes (which take up a length of 3 mm; see Figure 1) were positioned in the center of the tumor. As a conclusion, we might estimate that the electrical measurement might only be impacted by the surrounding tissues in certain situations involving tiny tumors (less than 10 mm) and on the order of 5%.

## 5. Conclusions

Electrical properties have shown potential in effectively differentiating between various types of in vivo and ex vivo tissue, such as metastasis, cholangiocarcinoma (CCA), hepatocellular carcinoma (HCC), hepatic cirrhosis, normal liver, and normal liver. These findings could lay the groundwork for subsequent research. The data gathered could help to advance bioelectric applications, potentially improving tissue diagnostics and liver treatments that use electrical fields. Future studies could gather more clinical samples to further categorize tissue types and conduct a thorough comparison of the dielectric properties across different human liver tissues. This method could pave the way for the precise identification of lesion types and stages in liver tissues.

## Figures and Tables

**Table 1:** Electrical conductivity (S/m) at the chosen frequencies.

**Tissue Type**	**Frequency (kHz)**	**Mean ± SD (S/m)**
In vivo normal (n = 15)	3	0.13 ± 0.06
	30	0.17 ± 0.06
	300	0.30 ± 0.12
	607	0.39 ± 0.11
	1000	0.49 ± 0.10
Ex vivo normal (n = 16)	3	012 ± 0.07
	30	0.16 ± 0.09
	300	0.26 ± 0.10
	607	0.34 ± 0.10
	1000	0.38 ± 0.08
In vivo tumor (n = 16)	3	0.41 ± 0.10
	30	0.45 ± 0.1
	300	0.57 ± 0.12
	607	0.69 ± 0.22
	1000	0.78 ± 0.24
Ex vivo tumor (n = 3)	3	0.27 ± 0.09
	30	0.30 ± 0.09
	300	0.38 ± 0.08
	607	0.45 ± 0.11
	1000	0.54 ± 0.14
In vivo cirrhotic (n = 3)	3	0.09 ± 0.01
	30	0.11 ± 0.02
	300	0.22 ± 0.02
	607	0.31 ± 0.009
	1000	041 ± 0.01
Ex vivo cirrhotic (n = 3)	3	0.16 ± 0.01
	30	0.18 ± 0.02
	300	0.29 ± 0.05
	607	0.35 ± 0.07
	1000	0.40 ± 0.08

**Table 2:** The mean values of the ratio of conductivity in four different tissues.

Frequency (kHz)	Ratio					
	Tin/Nin ^a^	Tin/Tex ^b^	Tex/Nin ^c^	Tin/Cin ^d^	Tex/Cin ^e^	Nin/Cin ^f^
	Mean	Mean	Mean	Mean	Mean	Mean
3	3.2	1.5	2.1	4.4	3.0	1.4
30	2.7	1.5	1.8	3.8	2.6	1.4
300	1.9	1.5	1.2	2.5	1.7	1.3
607	1.8	1.5	1.1	2.2	1.5	1.3
1000	1.6	1.4	1.1	1.9	1.3	1.2

^a^ Ratio between in vivo tumor versus normal tissue. ^b^ Ratio between tumor tissue in vivo versus ex vivo. ^c^ Ratio between ex vivo tumor versus in vivo normal tissue. ^d^ Ratio between in vivo tumor versus in vivo cirrhotic. ^e^ Ratio between ex vivo tumor versus in vivo cirrhotic tissue. ^f^ Ratio between in vivo normal versus in vivo cirrhotic tissue.

**Table 3:** Mean and standard deviation of electrical conductivity (S/m) for tumor tissue in vivo in 3 different types of tumor.

Frequency (kHz)	Tissue Type	n	Mean ± SD
3	CCA	2	0.35
	HCC	5	0.42 ± 0.13
	Metastasis	6	0.41 ± 0.08
30	CCA	2	0.38
	HCC	5	0.47 ± 0.15
	Metastasis	5	0.44 ± 0.08
300	CCA	2	0.53
	HCC	5	0.60 ± 0.17
	Metastasis	5	0.52 ± 0.04
607	CCA	1	0.70
	HCC	5	0.80 ± 0.30
	Metastasis	4	0.58 ± 0.10
1000	CCA	1	0.89
	HCC	5	0.88 ± 0.31
	Metastasis	5	0.67 ± 0.14

**Table 4:** Mean and standard deviation of electrical conductivity (S/m) for tumor tissue ex vivo in 3 different types of tumor.

Frequency (kHz)	Tissue Type	n	Mean ± SD
3	CCA	1	0.28
	HCC	6	0.23 ± 0.09
	Metastasis	6	0.33 ± 0.07
30	CCA	2	0.30
	HCC	6	0.25 ± 0.10
	Metastasis	6	0.36 ± 0.06
300	CCA	2	0.43
	HCC	6	0.34 ± 0.11
	Metastasis	5	0.42 ± 0.03
607	CCA	2	0.58
	HCC	6	0.41 ± 0.14
	Metastasis	4	0.51 ± 0.01
1000	CCA	2	0.73
	HCC	6	0.48 ± 0.17
	Metastasis	6	0.60 ± 0.02

**Table 5:** Individual patient and electrical conductivity data were measured at 1 MHz.

Patient’s Number	Disease	Tumor Size (mm)	Conductivity (S/m)				
			Cirrhotic Tissue	In Vivo Normal	Ex Vivo Normal	In Vivo Tumor	Ex Vivo Tumor
1	CCA	88 × 76 × 70	No	0.71	0.37	0.9	0.73
2	HCC	67 × 50 × 45	No	0.43	0.54		0.78
3	MET	8 × 6 × 6	No	0.43		0.5	
4	HCC	27 × 20 × 16	Yes	0.43	0.50	0.8	0.45
5	HCC	43 × 33 × 21	No	0.43	0.27		
6	HCC	19 × 19	No	0.43	0.74		
7	MET	35 × 25 × 31	No	0.43	0.31	1.1	0.63
8	MET	40 × 24 × 37	No	0.43	0.67	0.8	0.62
9	HCC	23 × 17 × 15	Yes	0.43	0.41	0.7	
10	MET	24 × 20 × 26	No	043	0.29	0.6	0.62
11	HCC	24 × 22 × 19	No				
12	HCC	33 × 25 × 22	Yes	0.40	0.32	0.5	0.28
13	MET	22 × 16 × 13	No	0.41	0.35	0.8	0.61
14	MET	26 (Diameter)	No	0.56	0.38	0.7	0.44
15	HCC	85 × 80 × 65	No	0.60	0.47	1.1	0.54
16	HCC	10 × 7 × 6	No	0.52	0.42	0.9	0.34
17	HCC	57 × 48 × 48	No	0.72	0.44	1.4	0.52
18	MET	34 × 32 × 24	No	0.45	0.35	0.7	0.56
19	HCC	10 × 1 × 1	No	0.47	0.04	0.9	0.44

HCC: Hepatocarcinoma; MET: Metastasis; CCA: Colangiocarcinoma.

## Data Availability

The datasets used and analyzed during the study are available from the corresponding author upon reasonable request. Any restrictions on the availability of materials or information must be disclosed to the editors at the time of submission. It should be noted that due to technical problems in data collection, some amounts of data were unusable and were eliminated.

## Abbreviations

HCC	Hepatocellular carcinoma
CCA	Cholangiocarcinoma
RF	Radiofrequency ablation
IRE	Irreversible electroporation
MET	Metastasis
Nex	Ex vivo normal tissue
Tex	Ex vivo tumor tissue
Nin	In vivo normal tissue
Tin	In vivo tumor tissue
